# Systemic Sclerosis and Primary Biliary Cholangitis: A Comprehensive Review of Two Overlapping Rare Entities With Insights on Diagnostics and Management

**DOI:** 10.7759/cureus.82008

**Published:** 2025-04-10

**Authors:** Hemang H Thakkar, Nissy V Mathew, Etikala P Reddy, Anusha L Cheetiyar, Varun Kommalapati, Aksa Mathew, Abirami Rajendiran, Raina Riyaz, Nixon Joseph, Abdullah H Obadi, Nazmi Vahora, Mariam Alamgir, Hossam T Ali

**Affiliations:** 1 Internal Medicine, Gujarat Medical Education and Research Society (GMERS) Medical College and Hospital, Sola, Ahmedabad, IND; 2 Medicine, Cambridge University Hospitals NHS Foundation Trust, Cambridge, GBR; 3 Medicine, Kamineni Academy of Medical Sciences and Research Center, Hyderabad, IND; 4 Medicine, Theni Government Medical College, Theni, IND; 5 Internal Medicine, Allegheny Health Network, Pittsburgh, USA; 6 Internal Medicine, Manchester University NHS Foundation Trust, Manchester, GBR; 7 Medicine, Vinayaka Mission's Medical College and Hospital, Pondicherry, IND; 8 Internal Medicine, Shadan Institute of Medical Sciences and Research, Hyderabad, IND; 9 Internal Medicine, St. George’s University, True Blue, GRD; 10 Medicine, Near East University, Nicosia, CYP; 11 Internal Medicine, Smt. B. K. Shah Medical College, Vadodara, IND; 12 Medicine, St. George’s University, True Blue, GRD; 13 Qena Faculty of Medicine, South Valley University, Qena, EGY

**Keywords:** antimitochondrial antibody, primary biliary cholangitis, primary biliary cirrhosis, scleroderma, systemic sclerosis

## Abstract

Primary biliary cholangitis (PBC) is an autoimmune liver disease of a chronic nature that can lead to liver cirrhosis, predominantly in females. PBC frequently coexists with other autoimmune diseases, such as systemic sclerosis (SSc), rheumatoid arthritis, systemic lupus erythematosus, and Sjögren’s syndrome. Despite variations in the literature, most studies have reported that a few PBC patients have SSc, especially the limited cutaneous subtype. Pathology of SSc includes microvascular affection and widespread fibrotic changes along with the autoimmune process. This narrative review aims to provide a comprehensive overview of the existing literature up to December 2024 regarding PBC, SSc, and overlap syndrome with emphasis on diagnostic points. Clinical manifestations can be significantly overlapping for both conditions. Thus, laboratory and histopathological investigations are necessary. The antibody profile is a cornerstone in such autoimmune diseases. While the antimitochondrial antibody (AMA) is considered specific for PBC, the presence of anticentromere antibody (ACA) highly suggests the concomitant presence of SSc. Several common pathologic mechanisms and triggers have been suggested for both diseases, and genes like HLA-DRB1, DQA1, STAT4, and IRF5 are shared between the two conditions. It is noteworthy that the prognosis and outcome of PBC cases are affected by the presence of SSc; for instance, the high liver-related PBC mortality decreases with the presence of SSc, although overlapping cases are at high risk of non-liver-related mortality. The overlapping cases comprise a clinical challenge for diagnosis and tailored management, although some promising medications are being investigated for both conditions, possibly due to common pathogenic mechanisms. Herein, we comprehensively review the available literature on PBC-SSc overlapping syndrome in terms of epidemiology, underlying pathophysiology, and clinical aspects.

## Introduction and background

Primary biliary cholangitis (PBC), previously known as primary biliary cirrhosis, is a chronic autoimmune liver disease that predominantly affects middle-aged women, causing progressive chronic liver cirrhosis due to gradual intrahepatic bile duct destruction, resulting in periportal inflammation and cholestasis. Prolonged hepatic cholestasis subsequently leads to cirrhosis and portal hypertension [1-3]. Biochemical changes with PBC include cholestasis, elevated liver enzymes, and positive autoantibodies. Histologically, the disease is characterized by lymphocytic infiltration and damage to biliary epithelial cells (BEC), for which advanced cases may require liver transplantation [4].

PBC frequently coexists with other autoimmune diseases, such as systemic sclerosis (SSc), rheumatoid arthritis (RA), systemic lupus erythematosus (SLE), and Sjögren’s syndrome (SS) [5]. SSc is a multisystem autoimmune disease that is characterized by immune dysregulation, resulting in widespread microvascular damage and fibrotic changes in the skin and internal organs. Overlap between PBC and SSc is observed in 1.4%-17% of cases, particularly in women with limited cutaneous SSc (lcSSc). This overlap is often associated with anticentromere antibodies (ACA) and generally results in a slower progression of liver disease compared to PBC alone [6].

Histological and laboratory studies suggest that PBC shares genetic and immunological pathways with SSc, though the precise mechanisms remain unclear [5]. Studies using Mendelian randomization (MR) have sought to clarify these relationships but have produced inconsistent findings due to limited sample sizes and potential confounding factors [5]. While both diseases are not common, co-occurrence points to overlapping risk factors and possible underlying pathogenic mechanisms. Overall, exploring the genetic links and causal mechanisms between PBC and SSc is critical for advancing clinical management and improving patient outcomes [7-9]. Therefore, we conducted this comprehensive review to discuss the possible relationship of co-occurrence of SSc with PBC in light of the available evidence.

## Review

Epidemiology of PBC, SSc, and overlap

PBC disproportionately affects middle-aged women, with a strong female preponderance at a ratio of 9:1 (female to male) [1]. It is variable in prevalence worldwide; however, the global incidence rate of PBC is documented to be between 0.23 and 5.31 per 100,000 people, and its prevalence is reported to be between 1.91 and 40.2 per 100,000 people [3,6,10-12]. SSc has a female predominance but is less prominent than PBC, with a global incidence between 1.78 and 23.57 per 100,000 people [6,11-13].

PBC and SSc represent a unique overlapping autoimmune disease associated with gender-specific differences. The association of these diseases is of special concern; the prevalence of PBC in patients with SSc was reported to be between 2% and 3%, which is higher than that of the general population [4,5,14,15]. The observed gender differences in this double-linked disease entity could be related to complex immunological mechanisms. Current studies have suggested that the X chromosome and sex hormone-related factors play a significant role in the observed predominance of both entities in women [6,11,12]. This epidemiological trend demonstrates that PBC should be screened in any female with SSc, especially those with limited cutaneous symptoms, and PBC in SSc should be treated as it is an overlap syndrome that clinicians need to be aware of to facilitate early diagnosis and proper management.

Underlying pathogenesis and triggers

**Figure 1:**
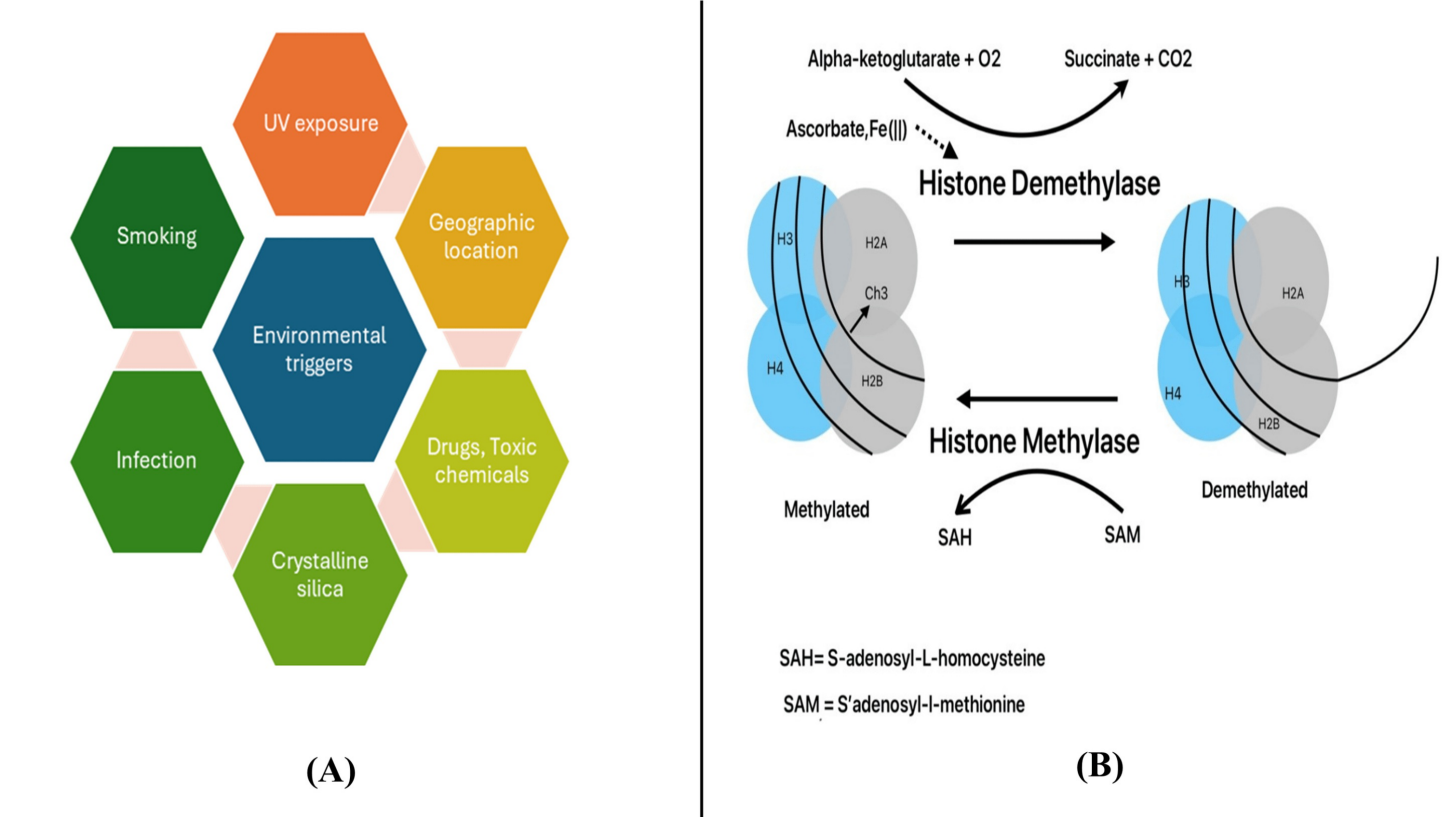
(A) Environmental triggers in PBC and SSc. (B) Fibroblasts demonstrate hypoacetylation of H3 and H4 histones at collagen suppressor gene promoters, contributing to excessive collagen synthesis and leading to fibrosis. The figure is an original creation by the authors. PBC: primary biliary cholangitis; SSc: systemic sclerosis.

Although rare, the association between PBC and SSc has been well documented. The widening overlap suggests that a combination of genetic and environmental exposures may predispose to a common pathogenic pathway of autoimmunity. Environmental triggers that have been studied in both diseases are summarized in Figure 1A [4,5,15]. Factors implicated in the pathogenesis of both diseases are illustrated in Table 1. Epigenetic modifications further influence the immune response in overlap syndrome. T cells show overexpression of JMJD3 histone demethylase in SSc patients, which activate genes like CD40L, CD70, and CD11a, which help in driving the autoimmune activation [16]. B cells exhibit histone modifications in SSc cases, such as methylation and demethylation, which correlate with the disease severity. Fibroblasts demonstrate hypoacetylation of H3 and H4 histones at collagen suppressor gene promoters, contributing to excessive collagen synthesis and leading to fibrosis (Figure 1B) [16-18].

**Table 1:** Factors involved in PBC and SSc pathogenesis. PBC: primary biliary cholangitis; SSc: systemic sclerosis; TGF-β: transforming growth factor-beta; IL: interleukin; TNFα: tumor necrosis factor-alpha; EBV: Epstein-Barr virus; CMV: cytomegalovirus.

Factor	Source/trigger	Role in PBC	Role in SSc
TGF-β [4,9,17]	Fibroblasts, immune cells	Promotes fibrosis and biliary epithelial cell injury	Promotes systemic fibrosis
IL-6 [9,19]	Immune cells	Drives inflammation and fibrosis	
TNFα [9,19]	Immune cells	Promotes disease progression and loss of self-tolerance	Type I collagen production by fibroblast and mediates vasculopathy and fibrosis
IL-2R [9,19]	Immune cells	Decreases Treg cells and promotes autoimmunity	Secretes immunomodulatory factors by stimulating epithelial and endothelial cells and fibroblasts
IL-17 [4,16]	Immune cells	Pro-inflammatory factor and induces inflammation	Alleviates immune and inflammatory response
CD40L [16]	Immune cells	Regulates T-cell autoreactivity	Promotes autoimmunity
Smoking [16,20]	Environmental	Increases oxidative stress and promotes autoimmunity	Enhances vascular damage and fibrosis
UV exposure [16,20]	Environmental	Triggers DNA damage and promotes autoimmunity	Induces oxidative stress and promotes autoimmunity
EBV, CMV [9,19]	Environmental	Triggers disease by molecular mimicry	Antibodies directed against viral protein UL94 cross reacts with NAG-2, expressed by endothelial cells and fibroblasts
HDAC7 suppression [16,21]	Epigenetic	Decreases type I/III collagen production and modulates T-cell autoreactivity	Modulates fibrosis and immune responses
H4ac [16,21]	Epigenetic	Induces CD40L and IL-17, suppresses the TNF-related apoptosis-inducing ligand (TRAIL), and regulates T-cell autoreactivity	Progression of disease activity

The pathogenesis of PBC is driven by an immune insult to the intrahepatic bile ducts, resulting in biliary epithelial cell (BEC) injury [19]. This process begins with the autoimmunity against autoantigen, along with BEC secreting cytokines during senescence. Pro-inflammatory cytokines cause the messenger ribonucleic acid (mRNA) to overexpress the E2 subunits of pyruvate dehydrogenase (PDH-E2) epitopes [20]. Pro-inflammatory cytokines also activate natural killer (NK) and dendritic cells. Dendritic cells, in turn, cause activation and differentiation of T cells. Notably, CD4+ and CD8+ T cells heavily infiltrate the portal tracts of affected individuals. These T cells contribute to BEC apoptosis through FasL expression, perforin secretion, and granzyme B release [3,4,21]. Chemokines like CXCL10, CXCL9, and CX3CL1 attract these T cells to the portal areas, where they exacerbate tissue damage. Additionally, CD1d-restricted natural killer T (NKT) cells are increased in PBC livers, linking the innate and adaptive immune systems [22]. The disease is further characterized by alterations in T helper (Th17) and regulatory T (Treg) cell populations. An increase in hepatic Th17 cells correlates with fibrosis progression, while a relative decrease in Tregs, particularly in the liver, is associated with the destruction of bile ducts. Notably, dendritic cells induce autoantibodies production by B cells. Apoptotic BECs fail to modify mitochondrial PDC-E2 with glutathione, preserving the lysine-lipoyl epitope recognized by circulating antimitochondrial antibodies (AMAs), leading to widespread immune activation and further BEC apoptosis [3,4,21]. These findings underscore the complex immune dysregulation in PBC, where innate and adaptive immune responses contribute to disease progression [22,23].

PBC is seen in 2% of patients having SSc, particularly limited cutaneous SSc and positive anticentromere antibodies [22]. SSc is a complex autoimmune disease marked by microvascular injury, immune system dysfunction, and progressive fibrosis that affects both the skin and internal organs [17]. While microvascular dysfunction is the disease's hallmark, research has demonstrated that large arteries are also impacted, leading to coronary artery disease and accelerated atherosclerosis, often occurring independently of traditional cardiovascular risk factors. The disease presents primarily in two forms: diffuse cutaneous SSc (dcSSc) and limited cutaneous SSc (lcSSc), which differ in the extent of skin involvement and associated organ complications [24]. At the core of SSc pathogenesis is the intricate interplay between vasculopathy and autoimmunity. Initial endothelial injury induces an immune response, leading to fibrotic and inflammatory changes in different tissues [25]. The disease process involves dysregulation of immune cells, particularly the activation of mucosal-associated invariant T (MAIT) cells and T cells, along with altered cytokine production (especially IL-6 and IL-13). This immune activity leads to changes in macrophages, particularly profibrotic M2-type macrophages, which further promote fibrosis through profibrotic cytokine release [26].

**Figure 2:**
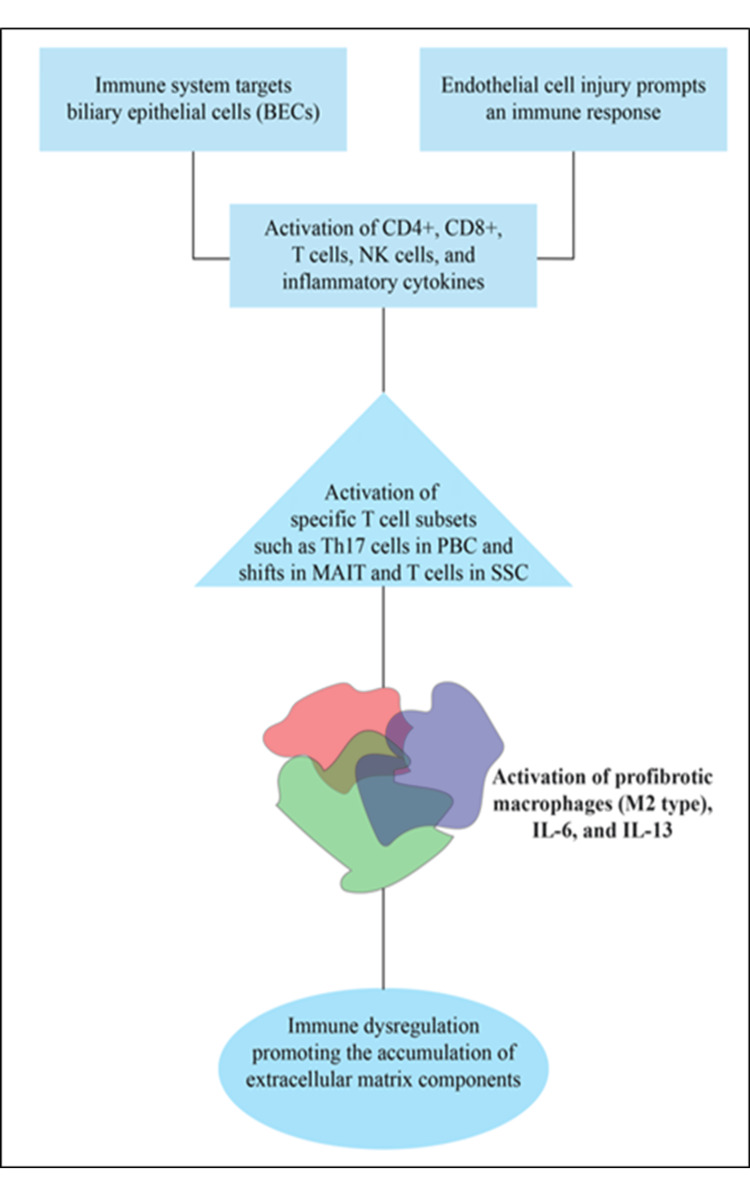
Common pathologic pathway in PBC and SSc. The figure is an original creation by the authors. BEC: biliary epithelial cells; CD: clusters of differentiation; NK: natural killer; PBC: primary biliary cholangitis; MAIT cells: mucosal-associated invariant T cells; SSc: systemic sclerosis; IL: interleukin.

Notably, both diseases involve innate and adaptive immune response dysregulation [27]. In PBC, innate immune responses, such as the activation of NK cells and the release of inflammatory cytokines, amplify the autoimmune process targeting BEC [28]. In SSc, dysregulated immune activity, including shifts in cytokine production (especially IL-6 and IL-13) and the activation of profibrotic macrophages, leads to tissue fibrosis and vascular damage [29]. Both conditions exhibit fibrosis as a central feature, with immune dysregulation promoting the accumulation of extracellular matrix components (Figure 2) [3,30].

Genetic background of PBC, SSc, and overlap

Both human leukocyte antigen (HLA) and non-HLA regions have been connected to PBC. Key HLA regions involved in PBC include DRB1, DQA1, DQB1, and DQA2. Non-HLA genetic regions associated with PBC include genes like IRF5, STAT4, SPIB, IKZF3-ORMDL3, IL12A, IL12RB, MMEL1, DENND1B, CD80, IL7, CXCR5, TNFRSF1A, CLEC16A, and NFKB1 [31-33]. In the largest combined genetic study to date, involving over 10,000 PBC cases from European and East Asian populations, researchers identified 22 additional genetic regions related to PBC risk, some of which were specific to certain populations [34]. It is understood that family members of an individual affected by PBC are at a 100-fold higher risk of developing PBC [35].

SSc was found to occur more frequently in families with a history of the disease compared to the general population [36]. In a related study, immediate family members who both had SSc were more likely than expected to share specific immune markers, such as certain autoantibodies and genetic markers within the HLA class II region [37]. Early genetic studies in SSc patients found the strongest link to disease risk within the HLA class II region on chromosome 6. However, due to variations in gene patterns across different ethnic groups and the complexity of the HLA system, pinpointing the exact gene involved has been challenging. Follow-up studies using additional methods have helped identify several other genes outside the HLA region that also influence SSc risk. Researchers have discovered several genetic regions outside the HLA system linked to SSc. These include genes like STAT4, IRF5, BANK1, TNFSF4, TBX21, IL-23R, and C8orf13-BLK, among others [38-46].

Although there is no common etiology between SSc and PBC, some genetic factors have been linked to various autoimmune diseases, although they are not exclusive to any one disease. Genes like HLA-DRB1, DQA1, STAT4, and IRF5 are shared between the two conditions [30,47]. Both PBC and SSc are associated with higher levels of certain proteins that promote fibrosis and are involved in the function of immune cells called T-helper (Th)-17 cells and regulatory T (Treg) cells, such as TGF-β (transforming growth factor) and IL-6 (interleukin 6) [30,48]. Research has shown that the way these immune cells work in PBC and SSc is quite similar. Overall, these genetic studies suggest that SSc shares a genetic basis with other autoimmune diseases, mainly through immune system dysfunction (Table 2).

**Table 2:** Genes that have been linked to PBC and SSc.

Disease	Gene	Role and importance
Primary biliary cholangitis (PBC) [33-35]	HLA-DQB1	Strongly linked to autoimmune liver diseases, contributing to antigen presentation abnormalities.
	IL12A	Involved in cytokine signaling. Promoting inflammatory responses and contributing to liver autoimmunity.
	SPIB	Associated with B-cell development and function, implicated in PBC-specific immune responses.
	CLEC16A	Plays a role in autophagy and immune regulation, linked to the breakdown of self-tolerance in the liver.
	DENND1B	Influences cytokine production and inflammatory signaling pathways specific to liver-targeted autoimmunity.
Systemic sclerosis (SSc) [40-48]	HLA-DQA1	Variants such as HLA-DQA1*0501 contribute to immune dysregulation and fibrotic processes.
	PTPN22	Regulates T-cell activation, with variants promoting excessive immune activity in SSc.
	NFKB1	Critical inflammatory signaling variants may drive chronic inflammation and fibrosis in SSc.
	IKZF1	Regulates immune cell development, particularly lymphocytes, involved in SSc-associated autoimmunity.
	CD247	Plays a role in T-cell receptor signaling, contributing to immune dysfunction specific to SSc.
Both PBC and SSc [33-35,40-48]	STAT4	Associated with immune signaling and cytokine regulation, common in various autoimmune diseases.
	IRF5	Implicated in the production of type I interferons, a shared pathway in both PBC and SSc.
	TYK2	Mediates cytokine signaling in the immune response, contributing to inflammation and fibrosis in both conditions.
	HLA-DRB1	Shared alleles influence antigen presentation and immune system tolerance in autoimmunity (HLA-DRB1*08 in PBC; HLA-DRB1*1104 in SSc).
	IL7R	Involved in T-cell development and survival, contributing to the breakdown of self-tolerance in both.

Histopathological features of PBC, SSc, and overlap

PBC and SSc are separate autoimmune diseases with distinct histological characteristics. However, certain histopathological and laboratory findings may be shared by various disorders when they overlap, which makes diagnosis and treatment more difficult. The hallmark of PBC is the gradual degeneration of the intrahepatic bile ducts, which results in cirrhosis and cholestasis [2,23]. Histologically, it is characterized by portal inflammation and lymphocytic infiltration, which frequently includes granulomas, especially in the early stages [3,27]. The liver exhibits fibrosis around portal sites, loss of bile ducts, and ultimately cirrhosis as the condition worsens. The inflammatory infiltration consists primarily of CD4+ T cells, macrophages, and B cells, with a slow progression from portal inflammation to fibrosis and lobular damage [15,49,50].

SSc is a multi-organ fibrotic condition that typically affects the skin, lungs, kidneys, and gastrointestinal tract. SSc can result in vascular alterations such as vasculopathy and vasculitis, bile duct injury, and periportal fibrosis in the liver [51,52]. Histopathologically, SSc-related liver disease is frequently linked with portal fibrosis and lobular abnormalities, small bile duct loss (similar to PBC), and a less significant inflammatory infiltration [53].

Histopathological characteristics of PBC and SSc, as well as their overlap, give key diagnostic insights. While SSc involves fibrosis and vascular alterations, frequently affecting numerous organs, PBC is essentially a biliary illness marked by loss of bile ducts and granulomas. The overlap syndrome combines characteristics of both illnesses to produce a heterogeneous histopathological appearance [54,55]. PBC histological characteristics in the liver, such as portal inflammation, ductopenia, and fibrosis, coexist with vascular sclerosis and periportal fibrosis found in SSc. The exocrine glands may also show a mixed pattern of lymphocyte infiltration, which is typical of both autoimmune diseases [54-56].

Clinical symptomatology of relevance to both and the overlap syndrome

Both PBC and SSc can cause severe impairment in patients. While each disease entity bears specific clinical features, there is also an overlapping symptomatology that may confuse diagnosis and management [3]. The main target of PBC is the liver, which is characterized by progressive destruction of the bile ducts. The typical symptoms of PBC include fatigue, which is often described as debilitating and chronic, making it one of the most common complaints among patients. Pruritus, or severe itching, is another frequently reported symptom that can significantly affect quality of life [4]. Xanthelasma, characterized by yellowish fat deposits under the skin, is present in a minority of patients. In the later stages of the disease, jaundice may develop as a result of bile obstruction, and this can lead to fat-soluble vitamin deficiencies and malabsorption. Additionally, many patients experience right upper quadrant abdominal pain, often linked to liver dysfunction, which may sometimes improve spontaneously [51,57]. These symptoms collectively highlight the impact of PBC on patients' overall health and well-being.

SSc is characterized by skin thickening and involvement of multiple organs [58]. The lcSSc is characterized by limited organ involvement, although some cases can experience isolated pulmonary hypertension [57]. Key clinical features include skin changes such as tightening, sclerodactyly, and telangiectasia, which are often early indicators of the disease. Raynaud’s phenomenon is another common feature, where patients experience episodes of vasospasm in response to cold or stress, causing color changes in the fingers and toes. Other systemic affections can be presented as interstitial lung disease, pulmonary hypertension, and esophageal dysmotility, leading to gastroesophageal reflux and dysphagia. Furthermore, many patients experience fatigue and weakness, which can result from the disease itself or as side effects of treatment [58-60].

Although the coexistence of PBC and SSc is well-documented, significant diagnostic difficulties exist mainly due to the overlapping signs and symptoms [51,59]. Shared features in both conditions include increased fatigue in patients, which can become particularly problematic to manage with the combined effects of the two diseases. Pruritus, a very characteristic symptom of PBC, may be exacerbated due to skin changes seen in SSc. Liver-related symptoms like jaundice and hepatomegaly are also shared since liver function and bile ducts may be impaired in both conditions. Gastrointestinal symptoms, including dysmotility, are very common and need special care. In some cases, telangiectasia of the fingertips and lips may give an appearance similar to that seen in Rendu-Osler-Weber syndrome [58]. These overlapping features necessitate a comprehensive approach to diagnosis and treatment. The clinical symptomatology of PBC and SSc is an important point to be taken into consideration for making a timely diagnosis and management (Table 3). Overlapping symptoms, including fatigue and gastrointestinal manifestations, undeniably indicate a comprehensive approach to patient care. Further research is necessary to fully clarify the pathophysiology of these associations to arrive at an improvement in therapeutic strategies [51,58].

**Table 3:** Clinical manifestations occurring in PBC and SSc. References [3,4,53,60-62]. PBC: primary biliary cholangitis; SSc: systemic sclerosis.

Clinical manifestation	PBC	SSc	Overlap
Fatigue	Very common, often debilitating	Very common, related to systemic inflammation	Fatigue is a prominent symptom in both conditions
Skin changes	Hyperpigmentation and dryness of the skin	Sclerodactyly, skin thickening	Concurrent skin changes may occur in overlapping cases
Pruritus (itching)	Common, due to cholestasis	May occur due to cutaneous involvement	May co-occur in patients with both conditions
Joint pain/stiffness	May occur due to chronic inflammation	Common, associated with fibrosis	Joint involvement in overlap cases
Gastrointestinal symptoms	Dyspepsia, diarrhea, steatorrhea	Esophageal reflux, dysphagia, and may experience chronic diarrhea with gastrointestinal (GI) tract involvement	Overlap patients often experience GI symptoms
Sicca symptoms	Occasional dry mouth and eyes	Common in limited cutaneous SSc	Shared features in overlap patients

Diagnostics and autoantibodies

PBC is diagnosed based on the biochemical criteria of raised serum alkaline phosphatase (ALP), liver histology showing cholangitis and destructive interlobular bile ducts, elevated IgM, raised aminotransferases, and the presence of highly disease-specific autoantibodies like AMA and anti-nuclear antibodies (ANA) (Sp100 and anti-glycoprotein-gp210) [19,61,62]. Skin biopsy, nailfold visualization, capillaroscopy, laser Doppler, thermography for Raynaud’s, ANA antibodies, and specific antibodies like anti-topoisomerase I (ATA), ACA, anti-RNA polymerase III (ARA), anti-Th/To, and anti-U3 RNP are the several diagnostic tests where the presence of SSc have to be suspected [20,63]. Autoantibodies are the traditional biomarkers that are usually produced in the patient much before the clinical onset of the disease and are used in the confirmation of the diagnosis of PBC and SSc. They can contribute by helping in the identification of at-risk individuals, timely diagnosis, phenotyping, as well as their prognostic significance and therapeutic implications [64].

The presence of AMA in the sera of PBC patients was considered to be an autoimmune marker specific to the disease for its prompt diagnosis, but its correlation with the severity of the disease has not been established to date [65]. AMA has been present in 95% of PBC and 25-30% of SSc patients, which strongly indicates mitochondrial reactivity, particularly to the 70 kD and 50 kD antigens, thus being a highly specific biomarker for the evaluation of PBC [66-68]. In a study conducted in 2009, PBC-specific autoantibodies were also detected in 15% of lcSSc patients [69]. While AMA by indirect immunofluorescence demonstrated a sensitivity of 62.5% and a specificity of 97.2%, anti-MIT3 antibodies by enzyme-linked immunosorbent assay (ELISA) demonstrated an overall higher sensitivity and lower specificity of 75% and 85.4%, respectively, for the diagnosis of PBC [23]. In a study where 817 patients with SSc were examined to identify cases with PBC, MIT3 ELISA was the test used to detect AMA, and it showed to have a sensitivity of 81.3% and specificity of 94.6% [3]. Sp100 antibodies were detected to have higher sensitivity (31.3%) and specificity (97.4%) than gp210. But when MIT3 and sp100 antibody tests were combined, there was an enhancement of sensitivity to 100%, whereas specificity dropped to 92.6%. The results of the study of 817 patients thus support the comparatively high frequency of PBC in SSc and finally imply that the relatively low sensitivity of AMA-MIT3 antibodies for PBC detection can be improved by using combined AMA-MIT3 and sp100 antibodies as a diagnostic tool [3].

As regards SSc, some specific autoantibodies will be produced; first of all, ACA (87%), ATA (51%), and ARA (30%), and all these are instrumental in disease pathogenesis [70-72]. Since they were described in 1980, ACA has been recognized to be diagnostic of SSc [61,73]. This is an autoantibody that is specific to the centromere region of chromosome [74]. Additionally, ACA was found to be associated with a phenotype that was an intermediate between primary and secondary SSc [65]. ACA and AMA can co-exist in up to 25% and 30% of patients with scleroderma and PBC, respectively [6]. Autoantibody profiles will further help in defining this overlap. ACA is highly prevalent in lcSSc (87%) and is strongly associated with an increased risk of PBC in 40-50% of patients because they are reactive to protein C [22,71,75-79]. AMA has been present in 95% of PBC and 25-30% of SSc patients [66-68]. PDC autoantibodies, such as those targeting the E1β subunit, are seen in 40-50% of PBC-SSc overlap cases [17,80], and E1α subunit antibodies in 15-20% of SSc cases, associated with PBC development [81]. Even after three years of follow-up, a broad spectrum of PBC antibodies was found in SSc without an increase in cholestatic liver enzymes, thus rectifying that there is a significant concern of overlap between the diagnostics of PBC and SSc [15]. These immune markers can help in diagnosing the immune overlap. There has been evidence for some serological immune markers like IgG and IgM anti-PDC antibodies in 30-40% of SSc cases, indicating a high probability for PBC development [81]. Table 4 shows the most significant antibodies in both conditions.

**Table 4:** Role of antibodies in the diagnosis of PBC and/or SSc. PBC: primary biliary cholangitis; SSc: systemic sclerosis; lcSSc: limited cutaneous systemic sclerosis.

Antibody	Role in PBC	Role in SSc	Overlap cases
Anti-mitochondrial antibody (AMA) [68-70]	Highly (95%) indicative of PBC	Present in 25–30% of SSc patients	Common in overlap cases; indicates mitochondrial reactivity
Anti-centromere antibody (ACA) [24,68,73,81-84]	Seen in 29% of PBC cases	Highly prevalent (87%) in lcSSc	Strongly associated with PBC-lcSSc overlap cases
Pyruvate dehydrogenase complex (PDC) autoantibodies [14,17,20,24,82]	Targets E1β subunit, highly specific for PBC (93%) diagnosis	E1α subunit antibodies are found in 15–20% of SSc cases	High reactivity to E1α, E1β, X/E3BP, and E2/E3 in 20–30% of overlap cases
IgG and IgM anti-PDC antibodies [83,85]	Elevated IgM levels (89%) are highly characteristic of PBC	Elevated anti-IgG PDC antibodies in SSc (18%)	High probability for PBC development in SSc

Management considerations of PBC, SSs, and co-existence

The general strategy for the treatment of PBC aims to slow the progression of the disease and to treat patient-specific symptoms [82]. So far, no treatments have been shown to reverse the disease. Ursodeoxycholic acid (UDCA) is considered the primary modality of treatment of primary biliary cirrhosis, and obeticholic acid is considered the second line of treatment [82,83]. Studies have shown survival benefits with the use of UDCA. UDCA has been shown to improve liver chemistries and survival-free liver transplantation, and delay histological progression. It has also been shown to delay progression to hepatic fibrosis in end-stage disease [4,84]. Despite the use of UDCA, a portion of patients still progress toward cirrhosis, requiring liver transplantation. There is evidence to suggest that the use of UDCA delivers comparable responses in both AMA-positive and AMA-negative patients with PBC [82,83]. This, along with the proposed mechanisms of action of UDCA, suggests that UDCA does not address the root cause of the disease that leads to bile duct destruction. Given the body of evidence in PBC that points toward the immunological etiology of the disease, several immunosuppressive medications such as corticosteroids, azathioprine, D-penicillamine, cyclosporin, methotrexate, and colchicine have been tried with no compelling evidence of benefit [21,58,82]. Apart from treatments geared toward slowing down disease progression, symptomatic treatment is given to patients with pruritus with the use of bile acid sequestrants such as cholestyramine [82]. Vitamin deficiencies and osteoporosis are treated with vitamin supplementation and bisphosphonates, respectively. Ultimately, patients who progress toward end-stage cirrhosis are considered for liver transplantation [4,85].

More recently, peroxisome proliferator-activated receptor (PPAR) agonists have also been shown to exhibit an immunomodulatory role in PBC. They reduce portal inflammation and T-cell numbers in portal tracts. FDA-approved PPAR agonists are elafibranor and seladelpar. There is evidence to show that elafibranor, a dual PPAR-alpha and PPAR-delta agonist, improves biochemical parameters of cholestasis in patients with PBC [86,87]. A study with 161 patients showed a superior biochemical response with elafibranor compared to placebo after 52 weeks [87]. Seladelpar, a PPAR-delta agonist, has also been shown to improve biochemical parameters related to cholestasis compared to placebo. When studied in a phase 3 double-blinded placebo-controlled trial of 193 patients, seladelpar showed superior outcomes. In this study, about 98% of patients received UDCA as standard of care in addition to either seladelpar or placebo; 61.7% of the seladelpar group showed biochemical response compared to 20% in the placebo group. In addition, 25% of the seladelpar group had normalization of alkaline phosphatase versus 0% in the placebo group [86].

As for SSc, due to the complexity of the disease and the multisystem involvement, a single treatment strategy cannot be applied to the treatment of the disease. The two aspects of the treatments are to first target the primary pathophysiological mechanism of the disease and second, to treat the complications relevant to the organ system involved. Treatments for early diffuse SSc include immunosuppressive agents such as mycophenolate, methotrexate, cyclophosphamide, rituximab, and tocilizumab [88]. These agents are predominantly of value in decreasing the progression of skin fibrosis and interstitial lung disease (ILD). Hematopoietic stem cell transplantation has also shown improvement in skin and stabilized lung function [89]. Antifibrotic agents such as nintedanib have been shown to be beneficial in patients with ILD associated with SSc, and pirfenidone possibly has value in the treatment [88,90]. Complications require specific interventions on a case-by-case basis. While many drugs are being studied for SSc, PPAR-gamma agonists have been shown to abrogate the TGF-beta-induced stimulation of collagen synthesis and myofibroblast differentiation in mouse models of SSc [91].

The coexistence of SSc along with PBC is challenging and not uncommon. Fibrosis seems to be the final common pathway in both diseases [91]. Potential drug targets could be the common underlying mechanisms, such as abnormal fibroblast function and regulation of profibrotic factors such as profibrotic transforming growth factor-β and interleukin 6 [9]. PPAR agonists have shown promising results when used in PBC, as noted in the prior section. There is evidence to suggest PPARγ has a role in regulating TGF-beta-dependent fibrogenesis. PPARγ agonists such as rosiglitazone and pioglitazone have been shown to reduce cell proliferation and increase apoptosis of fibroblasts. A review by Liu et al. [91] illustrates various studies, including a genome-wide association study (GWAS) follow-up study that suggested the role of PPARγ in SSc and pulmonary arterial hypertension (PAH). They concluded that, given the antifibrotic effects, PPARγ agonists could be drug targets for SSc [92]. Given the established role of the PPAR signaling pathway in both diseases, more research is warranted on this in patients with the coexistence of PBC and SSc.

Clinical implications on prognosis and outcomes

The co-existence of PBC and SSc presents a significant challenge for clinicians, making both diagnosis and treatment complex. Autoimmune diseases like PBC and SSc often co-occur more commonly in females and individuals with a family history of autoimmune disorders. Interestingly, co-existence has been associated with less severe conditions compared to PBC alone, although it can complicate diagnosis and management due to overlapping symptoms, which further impacts the overall prognosis [12,93]. Research indicates that individuals with both PBC and SSc have higher mortality rates from SSc, which has a mortality rate up to four times higher than the general population [9,58,94]. The coexistence of PBC and SSc also severely impacts the patient's quality of life. Studies have shown that chronic fatigue is prevalent in patients with both diseases, leading to psychological distress and functional impairment [69]. Additionally, reduced esophageal motility has been observed in some patients, contributing to gastrointestinal issues and further diminishing the quality of life [21,95].

Additionally, the presence of PBC in patients with SSc significantly heightens the risk of developing cancer, surpassing the risk associated with other factors such as old age and the presence of ACA. In one study, SSc patients with PBC were found to have a 2.35-fold higher risk of breast cancer compared to those without PBC [96,97]. While this increased cancer risk is notable, it does not always correlate with a higher overall mortality rate, as factors like organ failure and disease progression also play a significant role in patient survival. Furthermore, the presence of PBC in SSc patients has been linked to the occurrence of other autoimmune diseases such as SS, SLE, and RA, further complicating both diagnosis and treatment [52,57,97,98].

In patients with PBC-SSc overlap syndrome, liver-related mortality is lower than in those with PBC alone. For example, serum bilirubin levels in patients with PBC-SSc rise five times more slowly compared to PBC patients, suggesting a slower progression of liver disease [99]. However, this slower progression is countered by an increased incidence of non-liver-related deaths, such as those due to complications from SSc. PBC-SSc patients are more likely to suffer from infections like spontaneous bacterial peritonitis (SBP) and septicemia, which could be linked to immune system abnormalities and organ dysfunctions caused by SSc [58]. While liver disease progresses more slowly in these patients, the overall survival benefit is neutralized by the higher mortality associated with SSc, making the overall prognosis similar to that of patients with PBC alone [9,58].

Current prognostic models for PBC may not fully apply to patients with both PBC and SSc, underscoring the need for tailored models that account for the complexities of liver-related mortality and the potential need for liver transplantation in these populations. Further research into the interplay between these diseases is essential to better understand the factors contributing to improved outcomes for these patients. Developing distinct prognostic models for patients with both PBC and coexisting autoimmune conditions could lead to more accurate predictions and ultimately improve patient management and outcomes [58,74,77,82,100].

## Conclusions

While the concomitant presence of autoimmune diseases is not uncommon, the present review delves into the co-existence of PBC and SSc. However, considering limitations of the available literature, such as variable sample sizes, inconsistencies in genetics, and background of patients, along with different objectives of the studies, long-term real-world studies focusing on patients with such co-existence are needed to precisely estimate the outcomes and prognosis of such patients.
